# Exploring the use of mobile phone technology for the enhancement of the prevention of mother-to-child transmission of HIV program in Nyanza, Kenya: a qualitative study

**DOI:** 10.1186/1471-2458-13-1131

**Published:** 2013-12-05

**Authors:** Larissa Jennings, John Ong’ech, Rogers Simiyu, Martin Sirengo, Seble Kassaye

**Affiliations:** 1 Research Unit, Elizabeth Glaser Pediatric AIDS Foundation, 1140 Connecticut Avenue NW, Suite 200, Washington, D.C. 20036, USA; 2 Department of International Health, Social and Behavioral Interventions, Johns Hopkins Bloomberg School of Public Health, 615 N. Wolfe Street, Room E5038, Baltimore, MD 21205, USA; 3 Elizabeth Glaser Pediatric AIDS Foundation, ABC Place, 4th Floor Building 2, Waiyaki Way Westlands, P. O. Box 13612-00800, Nairobi, Kenya; 4 Kenyatta National Hospital, University of Nairobi, Hospital Road, P.O. Box 20723, Nairobi, Kenya; 5 National AIDS/STD Control Programme, Ministry of Health, P.O. Box 19361–00200, Nairobi, Kenya; 6 Department of Medicine, Division of Infectious Diseases, Georgetown University, 3800 Reservoir Road, 5th Floor PHC Building, Washington, D.C. 20007, USA

**Keywords:** Mobile phone, mHealth, Short message service, SMS, HIV, Prevention of mother-to-child transmission, PMTCT, Male involvement

## Abstract

**Background:**

Community-based mobile phone programs can complement gaps in clinical services for prevention of mother-to-child transmission (PMTCT) of HIV in areas with poor infrastructure and personnel shortages. However, community and health worker perceptions on optimal mobile phone communication for PMTCT are underexplored. This study examined what specific content and forms of mobile communication are acceptable to support PMTCT.

**Methods:**

Qualitative methods using focus groups and in-depth interviews were conducted in two district hospitals in Nyanza Province, Kenya. A total of 45 participants were purposefully selected, including HIV-positive women enrolled in PMTCT, their male partners, community health workers, and nurses. Semi-structured discussion guides were used to elicit participants’ current mobile phone uses for PMTCT and their perceived benefits and challenges. We also examined participants’ views on platform design and gender-tailored short message service (SMS) messages designed to improve PMTCT communication and male involvement.

**Results:**

Most participants had access to a mobile phone and prior experience receiving and sending SMS, although phone sharing was common among couples. Mobile phones were used for several health-related purposes, primarily as voice calls rather than texts. The perceived benefits of mobile phones for PMTCT included linking with health workers, protecting confidentiality, and receiving information and reminders. Men and women considered the gender-tailored SMS as a catalyst for improving PMTCT male involvement and couples’ communication. However, informative messaging relayed safely to the intended recipient was critical. In addition, health workers emphasized the continual need for in-person counseling coupled with, rather than replaced by, mobile phone reinforcement. For all participants, integrated and neutral text messaging provided antenatally and postnatally was most preferred, although not all topics or text formats were equally acceptable.

**Conclusions:**

Given the ubiquity of mobile phones in Kenya and current health-related uses of mobile phones, a PMTCT mobile communications platform holds considerable potential. This pre-intervention assessment of community and health worker preferences yielded valuable information on the complexities of design and implementation. An effective PMTCT mobile platform engaging men and women will need to address contexts of non-disclosure, phone sharing, and linkages with existing community and facility-based services.

## Background

Prevention of mother-to-child transmission (PMTCT) is a cornerstone strategy in reducing infant mortality from the human immunodeficiency virus (HIV). Yet, an estimated 330,000 children acquired HIV worldwide in 2011, over 90% through mother-to-child transmission [1]. Current strategies for preventing HIV infections in infants can reduce the risk of transmission from an estimated 30% to less than 2% [2]. Yet, in developing countries, low adherence and retention limit the utility of facility-based PMTCT services to ensure appropriate prophylaxis and treatment from pregnancy to delivery and continuing in the postnatal period until the cessation of breastfeeding [3]. Community-based approaches that engage families and equip health workers with supportive communication tools to improve PMTCT cascade completion remain a priority for global scale-up and sustainability [4].

This study describes findings from formative research to examine pre-intervention community and health worker perceptions on use of mobile phones to improve PMTCT-related communication. Given that Africa is the fastest growing mobile phone market [5], mobile phone technology may hold promise for improving completion of the PMTCT cascade on a large scale in areas with poor health infrastructure and personnel shortages [6,7]. Short message service (SMS) text messages offer a low-cost and relatively unobtrusive method for strengthening information exchange. In the context of HIV services, several mHealth pilot interventions have highlighted the potential of mHealth strategies to improve antiretroviral treatment adherence and access to health information, as well as reduce turnaround times for receipt of laboratory HIV test results [8-10]. However, in the context of PMTCT, questions remain on whether similar strategies can effectively assist HIV-positive pregnant women to adhere to antiretroviral-based strategies demonstrated to effectively prevent transmission to HIV-exposed infants. Studies exploring pre-intervention preferences for mHealth initiatives have found that individuals are willing and interested in mobile-based health promotion [11,12]. Yet, information is needed on what specific content and forms of mobile communication are required and acceptable to support PMTCT.

In addition, women’s decisions to participate fully in PMTCT are often influenced by the opinions of their male partners and other family or community members. Given that men’s participation in PMTCT programs has been shown to encourage more supportive behavior for HIV testing, maternal ARV prophylaxis, and safe infant feeding [13-15], we examine how mobile phone technology can be used to encourage male participation in PMTCT. Ultimately, this research was conducted as a preliminary study to inform the development of a cluster randomized trial on the effectiveness of mobile phones for support of PMTCT. The acceptability of mobile phone use for PMTCT and implications for intervention design among HIV-positive women and their male partners are discussed.

## Methods

### Study design

A qualitative study was conducted using focus group discussions (FGDs) with HIV-positive pregnant women, their male partners, and community health workers (CHWs), as well as in-depth interviews (IDIs) with facility-based nurses providing PMTCT services. Two district hospitals with existing PMTCT programs were selected from Kendu Bay and Rachuonyo Districts in Nyanza Province, Kenya.

### Population and context

Nyanza Province has the highest HIV prevalence in Kenya at 14% with an estimated HIV prevalence among pregnant women of 8% [16]. The Kenyan Ministry of Health estimates that over 18,000 women were identified as HIV-positive in Nyanza Province [17]. Antenatal care (ANC) coverage in Kenya is high (92%), while skilled birth attendance remains low (44%) [16]. Only a minority of HIV-infected women and their infants successfully complete the PMTCT cascade through use of maternal and infant antiretroviral therapy and safe infant feeding. For example, 65% of HIV-infected women were initiated on antiretroviral prophylaxis while only 20% of HIV-exposed infants were tested for HIV at 6 weeks [17]. An estimated 17.3 million Kenyans aged 15 and older own a mobile phone [18], and 86% and 92% of women and men, respectively, reported using a mobile phone in the last week primarily for sending and receiving SMS messages [19].

### Sample selection

HIV-positive postpartum women were purposively recruited to participate in the study by CHWs who were oriented on the study’s objective and selection criteria. Women older than 18 years of age who were currently enrolled in or had completed PMTCT less than two years prior were eligible for the study along with their male partner. All on-site or available PMTCT community- and facility-based health workers were selected from each of the two district hospitals and the surrounding catchment area.

### Data collection

An open-ended topic guide for women and their male partners was used to elicit information on five core themes: current mobile phone use and ownership, perceived benefits and challenges of using mobile phones for PMTCT, views on priority messaging for SMS promotion of PMTCT, optimal design of a mobile phone-based PMTCT communication platform, and mobile-promoted male involvement for PMTCT. To examine current mobile use and ownership, participants were asked about their access to phones, including examples of how they had used mobile phones previously for health-related purposes. They were also asked about current challenges that women, partners, or health workers face in supporting PMTCT and how mobile phones might be employed to mitigate those challenges. To explore views on priority messages, twelve gender-tailored SMS mock-ups were presented across PMTCT core behaviors. Six SMS mock-ups were tailored for women enrolled in PMTCT services, and six were tailored for their male partners. The SMS mock-ups were translated from English to Kiswahili and the local language, Luo, and back-translated. Participants were asked to read the mock-up in the language of their choice, and describe what they understood it to convey, including any recommendations. To examine platform design features, participants were asked how often, when, or how long they would like to receive SMS for PMTCT and in what languages, including preferences for SMS versus phone calling. To examine mobile-promoted male involvement for PMTCT, participants were asked how mobile phones could be used to encourage male participation in PMTCT. Facility-based nurses and CHWs were asked to review the SMS mock-ups and describe their envisioned role in the mHealth PMTCT platform.

A total of six FGDs were conducted: two with women enrolled in PMTCT, two with male partners, and two with CHWs. Four IDIs with nurses were also conducted. This represented a total of 45 participants: 17 HIV-positive postpartum women, 12 male partners, 12 CHWs, and 4 nurses. All of the FGDs and IDIs were led by three social scientists with experience conducting qualitative research. The sessions were audio recorded and conducted in English, Kiswahili, or Luo, as selected by the participants. Each session lasted approximately 90 to 120 minutes. Focus groups were selected to provide an interactive format to capture multiple views on each proposed thematic area as a result of the dynamics and discussion of each group. Following the session, information on age, education, marital status, employment status, phone ownership, and prior experience sending and receiving SMS were also collected. Several stakeholder meetings with PMTCT advisors and program staff were likewise conducted as part of the SMS mock-up and tool development process.

### Data analysis

FGDs were translated and transcribed verbatim into English. Data analysis of the narrative data was conducted in two phases using a thematic approach. First, during data collection, FGDs were synthesized in detail by the research team immediately after each session to guide subsequent discussions. The team debriefings also helped to determine when saturation had been achieved and no new information had emerged. In the second phase, a manual preliminary analysis of the narrative data aimed to assemble the responses according to the pre-set themes in the FGD topic guide, which were then refined according to emergent themes. The analysis’ culminating step was to highlight relevant quotes provided in the text to illustrate major findings.

### Data quality

To enhance the credibility of results, the research team compared findings from each of the study’s sub-groups and organized a verification meeting with program advisors to examine the extent to which the research captured internally valid and dependable information. This included a review of typed field notes and confirmation of translation and transcription files. Demographic and contextual data were also obtained to facilitate the transferability of findings to similar contexts. The study additionally aimed to ensure concepts of data quality in qualitative research concerning authenticity and fairness were addressed by including direct quotes and a diverse range of views, including varying points of interpretation.

### Ethics approval

This study received ethics approval by the Kenyatta National Hospital, University of Nairobi Ethics and Research Committee in Nairobi, Kenya. Written informed consent was obtained for participation in the study as well as audio recording of the discussion. Participants were also asked to keep all discussions confidential. Data collected as part of the study were not linked to individual or personal identifiers.

## Results

### Demographic information and mobile phone status

The background characteristics of the participants are shown in Table 1. The mean age of women and men was 26 and 33 years, respectively. The mean age of CHWs was 42. The majority of participants currently owned a mobile phone and had prior experience receiving an SMS text. About half of the men and a third of women reported sharing their mobile phone, although mobile phone sharing was less common among CHWs and nurses. Among the four nurses who were interviewed, all owned a sole-use phone and had experience receiving and sending SMS. The main themes of the study findings included: current mobile phone use, perceived benefits and challenges of using mobile phones for PMTCT, views on priority SMS messaging for PMTCT, platform user preferences, and mobile-promoted male involvement for PMTCT.

**Table 1:** Background characteristics of study participants

**Characteristics**	**HIV-Positive Women**		**Male Partners**		**CHWs**	
	**Number**	**%**	**Number**	**%**	**Number**	**%**
**Total**	17	100	12	100	12	100
Mean age (in years)	26	–	33	–	42	–
Education						
Primary school	9	53	7	58	2	17
Secondary or more	8	47	5	42	10	83
Mobile phone access						
Sole owner	8	47	4	33	8	67
Shared	6	35	6	50	2	17
None	3	18	2	17	2	17
Ever received SMS text	15	88	10	83	10	83
Ever sent SMS text	12	71	9	75	10	83

### Current health-related mobile phone Use

Mobile phones are currently being used for a range of health-related purposes, although primarily in the context of voice calls. Calling health providers or CHWs to ask questions, receive assistance, or arrange an appointment was commonly mentioned by women. In some cases, this meant sending an SMS request for the health worker to call-back. Other health-related current uses were calling a neighbor or friend to seek assistance for labor and delivery, emergency transport to a health facility, or picking up medicines from the health center. Women also reported using phones to set-up or inquire about meetings within their PMTCT support groups or place a reminder on their phones for appointments.

“*We use phones to find out how our group members are doing. It is easier to reach me using a phone and also to know about my other group members’ welfare.” –* Woman, Kendu Bay

Men reported health-related uses of mobile phones primarily in response to their partner’s or family’s health needs or as a result of communication requests from CHWs. In some cases, mobile phones were used likewise to remotely support women during facility health visits.

*“I use it to remind my wife to take her drugs [and] also to remind her to give the child drugs especially during mornings because sometimes she can get very busy and forget.” –* Man, Kendu Bay

*“We use it for communication. Instead of coming all the way to seek for information, we just call. It is easier and cheaper to communicate on [a] phone. If my wife gets to the facility and an issue arises, she is able to call me back and tell me what is happening.” –* Man, Rachuonyo

Mobile phones supported CHW roles in coordinating attendance for community health action days or locating patients who had missed an appointment, as requested by a facility-based provider. CHWs also indicated that clients often called or sent SMS texts to them in order to reach a nurse for help. In some cases, CHWs also contacted fellow CHWs to refer difficult clients or seek additional support.

“*As for me, I call and also send SMS. Some people send ‘please call me’ [SMS texts] because they have something to say but do not have the airtime to call.” –* CHW, Kendu Bay

*“You can even book appointments through the phone. [Such as]… I’ll be coming to see you tomorrow. Kindly make yourself available. This is sent to both couples so that even if they do not share [a phone], they will both understand the purpose of the visit when you show up.”* – CHW, Rachuonyo

Nurses confirmed relying on CHWs to phone missing clients. In some cases, calls were made directly by a nurse as a result of recent efforts to document mobile phone contact information from patients during facility visits.

### Mobile opportunities and challenges for PMTCT

There were several perceived benefits of using mobile phones for PMTCT support. Women welcomed the remote access to health workers and ability to request additional antiretroviral drugs via phone which did not require them to travel to a health facility or risk disclosure by discussing their serostatus in an audio-permeable space at the health facility. Mobile phones also facilitated women’s and CHW’s ability to refer fellow HIV-positive women to a CHW within the area. CHWs also felt mobile phones enabled them to quickly notify clients about appointments or to reschedule client visits if drugs or supplies were unavailable. This was perceived as an important mechanism for saving time and money from unnecessary trips for both the client and the CHW.

“*The way I use my phone, for example: one day my husband was adamant not to be counseled. So, I called the counselor to speak to my husband who was sick and had refused to take his drugs.” –* Woman, Rachuonyo

“*Where are you? Why didn’t you come to the clinic? I ask them. [My] work is easier because I can consult many people if I have sufficient airtime.” –* CHW, Kendu Bay

The most common disadvantages for using mobile phones for PMTCT purposes included lack of sufficient funds for purchasing airtime or charging phones if there were multiple calls or SMS to be initiated. Mobile phones were valued for potential avoidance of unintended disclosure during in-person facility visits. However, several participants highlighted challenges in maintaining privacy when discussing a sensitive matter via a call or SMS. They also described experiences with delays in mobile-based communication if the recipient’s phone was not powered. Other disadvantages included uncertainty regarding how well voice or text messages would be received by individuals at a given time, including the perceived limitations of mobile communication to relay complex messages. Providers also noted challenges in verifying that the recommended task was completed if in-person visits were replaced by mobile communication.

*“I receive SMS, but I do not send. After reading an SMS I call back because it creates an opportunity to expand on a topic in detail. That way we can easily get into an understanding with the person on the other end.” –* CHW, Kendu Bay

*“Sometimes you may not know the mood of the person you are sending the message to. He may give a negative response if he is in a bad mood. With phones you cannot really trust the response you get from the other end.” –* Man, Rachuonyo

“*Responses [delivered by phone] could be untrue. They can tell you that they are doing it, but in a real sense nothing is being done*.”– CHW, Kendu Bay

### SMS promotion of PMTCT topics

The gender-tailored SMS mock-ups for PMTCT are shown in Table 2. For SMS tailored towards women, suggestions included encouraging women to take the initiative to request specific services at the health facility such as receipt of extra drugs or early infant testing, as well as explaining the benefits and risks of exclusive breastfeeding versus mixed feeding. Recommendations also included more integrated messaging for emergency preparedness and postnatal visits, in addition to supportive messaging for women who decided to engage their spouses for PMTCT. For SMS tailored towards men, suggestions included incorporating more messages relating to family pride and community recognition as a male role model. Nurses suggested likewise that SMS tailored for men provide information on the purpose of infant prophylaxis and benefits of communicating directly with health providers. Overall, the SMS mock-ups were perceived as relevant and encouraging by women, and men considered them to be empowering and persuasive.

*“This message makes a man feel valued. It makes him feel appreciated and immediately think of the wife and even increases love. It creates awareness… [and] brings more joy at home since it is likely to open up discussions and will make the wife happy.” –* Man, Rachuonyo

*“It’s a reminder, but I would also feel nice since it’s a sign that the nurses want to support you. The feeling of dying and giving up disappear because I will feel very encouraged and motivated that there is a concern for the status of my child.” –* Woman, Kendu Bay

**Table 2:** Mock-up SMS texts for women and men

**PMTCT Theme**	**Women’s SMS Mock-up**	**Men’s SMS Mock-up**
*ANC attendance and facility-based births*	For your good health and that of your unborn baby, attend ANC early and complete all appointments as advised by your health care provider. Always put money aside for hospital delivery.	Pregnancy is a man’s gift. Love your family, and accompany your spouse to the clinic during and after pregnancy. You will be an outstanding example.
*Early infant diagnosis*	Your baby’s first clinic appointment at 6 weeks is coming soon. Your baby will receive an important test. Try not to forget! If your baby has already been tested, have you received the results?	Your baby’s first clinic appointment is at 6 weeks. Book the date with your spouse. Your baby will receive an important test. You will also receive information to keep him/her healthy and strong. Try not to forget!
*Retention, adherence, and loss-to-follow-up*	Don’t stop taking your medicines and giving the baby his/hers. Go to the clinic if you are about to run out of medicines. Skipping doses makes medicines less effective over time. Stay connected with your provider!	Show others what good health is. Support your spouse in attending all her appointments during pregnancy and after birth. Don’t let her skip any important medications. She will be glad for your support. You will have a healthy mother and healthy baby!
*Exclusive breastfeeding*	Give your baby only the very best, your breast milk ONLY and no other foods or liquids for the first 6 months. Be strong against pressure. This will make your baby healthy and strong. Go to the clinic if you are having trouble with feeding.	As a new father, be sure your baby receives the very best. Encourage your spouse to breastfeed exclusively with no other foods or liquids. Exclusive breastfeeding will make your baby healthier. Everyone will notice his/her charm! If there are problems, visit the clinic for advice.
*Family planning*	Talk openly with your spouse to plan for your next birth. Use a modern family planning method that is suitable to both of you. Seek advice from the health care provider.	Be a smart guy. Talk openly with your spouse about planning for your next birth. Use a condom, and support your spouse to use any other modern family planning method.
*Partner involvement*	Make a birth plan with your spouse. Encourage him to take the test with you. Always ask him to accompany you to the ANC clinic. A health care provider will be pleased to talk to you both.	Family health starts with you. Know your status and keep yourself healthy. Talk with your spouse too. Your partner’s status is not always the same as yours. Together, you can support each other.

However, not all SMS topics were found to be equally acceptable. Among women, messaging on antenatal care and facility-based births as well as partner involvement and testing were most commonly mentioned as the most important SMS to be disseminated. Reasons cited were that engaging men early in pregnancy to provide support was likely to be more effective in improving women’s involvement and adherence to antenatal and PMTCT care services. Messages on family planning were perceived as less important among women given the relative urgency of breastfeeding, adherence, and early infant testing during the postpartum period. Responses were similar among men who indicated that partner ANC accompaniment and step-wise involvement would likely reduce women’s loss to follow-up over time. Messages on family planning were perceived as less suitable for mobile communication, as SMS or voice calls, among men.

### Optimal design of PMTCT mHealth platform

Women and men requested different frequencies to receive SMS messages depending on whether they shared a phone. Daily SMS message receipt was acceptable, but was preferred only a few times each week if the owner of the phone, such as a husband or neighbor, was traveling. There were also preferences for personalized rather than generic messages from health workers. Women requested as well the option to specify their language preference and select an optimal time of day to receive messages. Men tended to favor receipt of fewer messages delivered later in the day, while women were interested in more frequent messages at the start of the day with the possibility of suspending messages if the owner of the phone, usually a spouse, was traveling.

“*Once a day on a daily basis [to receive PMTCT SMS].Twice a day is good. Why should one get tired? Reading is not such a hard task.” –* Woman, Kendu Bay

*“As long as there is no charge, the messages can be sent as long as possible. [But]… one can get tired if you get a text on a daily basis. A few messages which are different each week are okay.” –* Man, Rachuonyo

Among health workers, the optimal platform was described as one in which face-to-face counseling by nurses or CHWs was reinforced using SMS, rather than replaced. Nurses indicated that time and resources to manage the platform could be challenging, although CHWs volunteered to enroll and orient clients as part of their current role.

***“*** *I would be pleased to hear a woman say, ‘I got your text’. I would then explain it in more detail. This makes counseling easier.” – CHW, Kendu Bay*

*“I must talk to the person first and get to know them. Then if I have a message for her, then I just send it. We can give out the [SMS] messages. Then we [would] do a follow-up to know exactly if both [the woman and her spouse] have received the information.”* – CHW, Rachuonyo

All respondents preferred a platform with SMS and phone call applications tailored for different purposes since SMS were perceived as advantageous for brief and relatively confidential receipts of information. On the other hand, phone calls were perceived to provide more opportunity for discussion and ensured the message was well received by the intended recipient with an immediate response.

### Mobile-promoted male involvement for PMTCT

Men and women perceived the gender-tailored SMS as motivational for improving couple’s communication regarding PMTCT and defining men’s role in PMTCT. Women felt that SMS sent to male partners could be used to initiate discussion, or if sent to women themselves, could be used to display key topics discussed during antenatal or postpartum clinic visits. In some cases, the tailored text messages were viewed as providing reminders and examples of persuasive wording for engaging men in PMTCT.

*“I would talk it out with my husband… It would encourage me to be free with my husband and make it easier to initiate dialogue because I can discuss the message with him. I would show him the message to trigger the conversation… We will discuss it so it’s not so strange for him.” –* Woman, Kendu Bay

*“This is a good message. If a man gets a message, the woman will find it even easier since she will just be reminding the husband about it.” –* CHW Kendu Bay

In addition to specific PMTCT-promoted behaviors, several men offered to forward SMS to other men as a means of affirming men’s PMTCT role. Mobile-based information was envisioned to allow men to send tailored messages to other male peers and empower them to get involved in partner or infant testing as well as other PMTCT-related behaviors.

*“I would send it to a friend in a similar situation. I would send [it to] him so that he can see me accompanying my wife. If he receives it, he can also benefit. Maybe, I will talk with him first… since a man who does not know about this could easily ignore the message thinking they are being cornered and would delete the message.” –* Man, Kendu Bay

*“When I get such a text, I would want to be an example. If a man has been doing this, he gets an encouragement that what he has been doing is right. It can trigger a man to continue supporting the family. You know…, when the children and family see that you are concerned, they appreciate you as a father.” –* Man, Rachuonyo

At that same time, participants suggested that two sets of messages be developed to be used for women and their partners in cases of serostatus disclosure and non-disclosure to avoid risks to women whose partners were unaware of their HIV serostatus. There was also recognition that SMS messages for men, in particular, would need to be linked with in-person communication, as initiation or by follow-up, in order to ensure that the message was welcomed and understood.

*“It can open discussions in cases where there is no disclosure, but it is good to disclose first. It depends if someone has disclosed such to the husband. If he does not know…, he might wonder why you are educating yourself only.” –* Woman, Kendu Bay

*“Most men will ignore. It could bring chaos before the man settles down for some discussion.” –* Man, Rachuonyo

## Discussion

Early programmatic experiences using mHealth platforms suggest a significant potential to address HIV care challenges using the expanding technology. However, health worker, women and men’s perceptions on optimal PMTCT mobile phone messaging have been underexplored. This study asked HIV-positive women receiving PMTCT services as well as their male partners and health workers to discuss their current use of mobile phones and their views on how best to structure a PMTCT-focused mHealth intervention engaging men and women, including review of several gender-tailored PMTCT SMS mock-ups.

The findings suggest that a PMTCT mHealth platform would be welcomed by couples and health workers, but should incorporate SMS messaging that is linked with in-person consultations with nurses and CHWs. Current mobile ownership and SMS literacy among participants suggested likewise that the majority of women, men, and health personnel could participate on the platform. Study participants were enthusiastic of the platform’s potential to surmount barriers of stigma or privacy loss which may occur during face-to-face counseling. They also favored the prospect of having routine text-based encouragement and supportive information for PMTCT.

However, given the variation of responses, the study highlighted several implications for designing an optimal platform. Automated SMS health messaging may need to be customized according to user preferences for language, timing, and scope of information provided. For example, users could select the topics on which they would like to receive more information, at what time of day, and at what frequency. This would enable women with variable phone access or HIV disclosure status to better manage the information provided.

At the same time, findings suggest that a standardized orientation process would need to be developed for all potential PMTCT mHealth enrollees. The orientation would enable health workers to verify women’s mobile phone numbers and their ability to initiate, send, and receive phone calls and SMS. The orientation would also provide an opportunity for women to consent to receipt of different types of PMTCT-related information. This was deemed critical by several stakeholders given the inherent challenge of developing distinct PMTCT-related messages which would not generate suspicion among unintended viewers, particularly male spouses in cases of non-disclosure. Enrollees would also need to have the option of suspending and reactivating service at their discretion, or enrolling two or more peers rather than their male partner. Finally, concerns among nurses regarding the overall management of the platform (since some clients may abuse the service) suggests that the platform would benefit from a central toll-free clinic phone or call center-based hotline for which enrollees are sensitized on when and how to use.

Interestingly, the study found that not all PMTCT topics were ranked equally. Women, men, and health worker respondents placed greater emphasis on messages promoting antenatal care and facility-based births as well as partner involvement to promote male testing and serostatus disclosure. Family planning messages were least valued. Our findings suggest this resulted because family planning norms were perceived more difficult to address via SMS or voice calls, and represented one of the more distal behaviors promoted across the SMS topics.

Preferences for male involvement SMS may reflect women’s and health workers’ desires to address partner disclosure and promote partner support for aspects other than PMTCT. Our findings suggested that having SMS tips or reminders was seen as a catalyst to initiate dialogue with male partners. Men also seemed eager to be recipients of PMTCT SMS messaging relating to their health and that of their families, and valued the role of forwarding motivational or informative texts to other men. At the same time, it was recognized by men, women, and health workers that mobile-based PMTCT communication targeting men may not always be welcomed, and would likely need a precursor or follow-up counseling session to be effective. Nonetheless, the potential of mobile communication to assist in defining men’s role in PMTCT should be further explored, as should the impact of integrating mobile-based PMTCT communication on mother-infant care and treatment. Overall, this study found that a safe, comprehensive, and gender-tailored platform with voice and SMS components linked to existing in-person PMTCT services would be acceptable among intended participants.

### Limitations

The limitations of this study deserve mention. The study did not explore current PMTCT communication received by women or men via existing media such as brochures, radio, women’s groups, or facility-based counseling. This may have provided greater insight on use of mobile technology to complement current gaps in PMTCT communication. Study participants were also drawn from communities who were actively engaged in the PMTCT program near district hospitals and may not represent couples in more remote areas with differential PMTCT outreach services, mobile phone literacy, or disclosure status. Individuals lost to follow-up were also not included.

In addition, because the study was designed as pre-intervention formative research, the largely positive findings may reflect a courtesy bias of participants’ envisioned response, rather than actual experiences, or responses to the researcher’s role in guiding the discussion and developing future PMTCT support services. To counter this, we asked participants to describe expected challenges and user concerns, including opinions which were not supportive of a mobile-based PMTCT platform. Implementation research will be needed to examine real user experiences and platform PMTCT effectiveness following its introduction among men and women.

Information is needed likewise to validate the safety and neutrality of proposed SMS messages among HIV-negative populations. The study’s SMS mock-ups were aimed to be direct enough to be recognizable to the intended recipient, but ambiguous enough not to generate suspicions or disclose the HIV status of the intended recipient in the event that the message was intercepted. This was examined hypothetically, but a more in-depth analysis would be needed in practice and across serostatus groups. The limited number of characters each SMS can obtain may also hinder effective communication. Finally, loss-to-follow-up among PMTCT service users is attributable to additional health systems, economic, and cultural factors, many of which are not addressed by mobile-based communication. This study highlights opportunities best linked to communication factors.

## Conclusions

Given the widespread coverage of mobile phones and current health-related uses of mobile phones in Kenya, a PMTCT mobile communications platform holds considerable potential. This study qualitatively examined community and health worker views on the content and design of a potential communication platform. The study’s pre-intervention assessment yielded valuable information on the acceptability of the planned intervention, as well as the complexities of design and implementation. Our findings highlighted that while women, men, and health workers perceived that tailored SMS and voice calls could improve PMTCT follow-up and men’s participation, a fully effective platform would also need to be customized to address contexts of non-disclosure, phone sharing, and linkages with existing community and facility-based counseling.

## Abbreviations

ANC: Antenatal care; ARV: Antiretroviral; CHW: Community health worker; FGD: Focus group discussion; HIV: Human immunodeficiency virus; IDI: In-depth interview; PMTCT: Prevention of mother-to-child transmission; SMS: Short message service.

## Competing interests

The authors declare that they have no competing interests.

## Authors’ contributions

LJ conceived and designed the study, developed the data collection instruments, participated in data collection, analyzed the results, and wrote the manuscript. JO assisted in interpreting study results and reviewing the study tools and manuscript. RS reviewed and finalized the data collection instruments, led the data collection, and assisted in the review of study results. MS reviewed data collection instruments and assisted in finalizing interpretation of study results and the manuscript. SK provided technical assistance in review of the analyses and made contributions to the manuscript. All authors have read and approved the final manuscript.

## Pre-publication history

The pre-publication history for this paper can be accessed here:

http://www.biomedcentral.com/1471-2458/13/1131/prepub

## References

[B1] Global Report: UNAIDS Report on the Global AIDS Epidemic Joint United Nations Programme on HIV/AIDS (UNAIDS) 2010 http://www.unaids.org/documents/20101123_GlobalReport_em.pdf

[B2] Townsend CL Cortina-Borja M Peckham CS de Ruiter A Lyall H Tookey P Low rates of mother-to-child transmission of HIV following effective pregnancy interventions in the United Kingdom and Ireland, 2000–2006 AIDS 2008 22 973 981 10.1097/QAD.0b013e3282f9b67a 18453857

[B3] Towards universal access: scaling up priority HIV/AIDS interventions in the health sector. Progress Report 2009 World Health Organization, United Nations Children’s Fund http://www.who.int/hiv/pub/tuapr_2009_en.pdf

[B4] Brown T Bukenya G Ostfeld S Empowering health workers with new technologies to end pediatric HIV/AIDS Global AIDS Alliance 2009 http://aidsalliance.3cdn.net/fc361fa6298013c139_flm6bxttf.pdf

[B5] GSMA Sub-Saharan Africa Mobile Observatory 2012 2013 GSMA. Deloitte. Pgs. 1–85. http://www.gsma.com/publicpolicy/wp-content/uploads/2013/01/gsma_ssamo_full_web_11_12-1.pdf

[B6] Chang LW Kagaayi J Nakigozi G Packer AH Serwadda D Quinn TC Gray RH Bollinger RC Reynolds SJ Responding to the human resource crisis: peer health workers, mobile phones, and HIV care in Rakai, Uganda AIDS Patient Care STDS 2008 22 3 173 174 10.1089/apc.2007.0234 18290750 PMC2674572

[B7] Lester RT Gelmon L Plummer FA Cell phones: tightening the communication gap in resource-limited antiretroviral programmes? AIDS 2006 20 2242 2244 10.1097/QAD.0b013e3280108508 17086071

[B8] Puccio J Belzer M Olson J Martinez M Salata C Tucker D Tanaka D The use of cell phone reminder calls for assisting HIV-infected adolescents and young adults to adhere to highly active antiretroviral therapy: a pilot study AIDS Patient Care STDS 2006 20 6 438 444 10.1089/apc.2006.20.438 16789857

[B9] Lester R Ritvo P Mills E Kariri A Karanja S Chung MH Jack W Habyarimana J Sadatsafavi M Najafzadeh M Marra CA Estambale B Ngugi E Ball TB Thabane L Gelmon LJ Kimani J Ackers M Plummer FA Effects of a mobile phone short message service on antiretroviral treatment adherence in Kenya (WelTel Kenya1): a randomized trial Lancet 2010 376 9755 1838 1845 10.1016/S0140-6736(10)61997-6 21071074

[B10] Pop-Eleches C Thirumurthy H Habyarimana J Zivin JG Goldstein MP de Walque D MacKeen L Haberer J Kimaiyo S Sidle J Ngare D Bangsberg DR Mobile phone technologies improve adherence to antiretroviral treatment in a resource-limited setting: a randomized controlled trial of text message reminders AIDS 2011 25 6 825 834 10.1097/QAD.0b013e32834380c1 21252632 PMC3718389

[B11] Crankshaw T Corless I Giddy J Nicholas PK Eichbaum Q Butler LM Exploring the patterns of use and feasibility of using cellular phones for clinic appointment reminders and adherence messages in an antiretroviral treatment clinic, Durban, South Africa AIDS Patient Care and STDS 2010 24 11 729 734 10.1089/apc.2010.0146 21039181

[B12] Shet A Arumugam K Rodrigues R Rajagopalan N Shubha K Raj T D’Souza G De Costa A Designing a mobile phone-based intervention to promote adherence to antiretroviral therapy in South India AIDS Behav 2010 14 3 716 720 10.1007/s10461-009-9658-3 20054634

[B13] Msuya SE Mbizvo EM Hussain A Uriyo J Sam NE Stray‒Pedersen B Low male partner participation in antenatal HIV counselling and testing in northern Tanzania: implications for preventive programs AIDS Care 2008 20 6 700 709 10.1080/09540120701687059 18576172

[B14] Reece M Hollub A Nagami M Lane K Assessing male spousal engagement with prevention of mother‒to‒child transmission (pMTCT) programs in western Kenya AIDS Care 2010 22 6 743 750 10.1080/09540120903431330 20461572

[B15] Aarnio P Olsson P Chimbiri A Kulmala T Male involvement in antenatal HIV counseling and testing: exploring men’s perceptions in rural Malawi AIDS Care 2009 21 12 1537 1546 10.1080/09540120902903719 20024733

[B16] Kenya National Bureau of Statistics (KNBS) and ICF Macro. 2010 Kenya Demographic and Health Survey 2008–09 Calverton, Maryland: KNBS and ICF Macro http://www.measuredhs.com/pubs/pdf/FR229/FR229.pdf

[B17] 2009 HIV Service Statistics: based on in-depth review of HIV Data Sources, February-June 2010 National AIDS & STI Control Programme M&E Department Report 2010 Kenya Ministries of Health

[B18] Kenya Economic Update. Still standing: Kenya’s slow recovery from a quadruple shock 2009 1 http://siteresources.worldbank.org/INTKENYA/Resources/ Kenya_Economic_Update_Dec_2009-full.pdf

[B19] Boyen H Information at the Grassroots: Analyzing the media use and communication habits of Kenyans to support effective development Audience Scapes Kenya: Africa Development Research Series http://audiencescapes.org/sites/default/files/AudienceScapes%20Kenya%20 Survey%20Research%20Report%202010.pdf

